# Construction and validation of a prognostic nomogram in metastatic breast cancer patients of childbearing age: A study based on the SEER database and a Chinese cohort

**DOI:** 10.3389/fonc.2022.999873

**Published:** 2022-11-25

**Authors:** Xiang Ma, Yapeng Xing, Zeying Li, Shun Qiu, Wenzhao Wu, Jinfeng Bai

**Affiliations:** Yunnan Cancer Hospital, The Third Affiliated Hospital of Kunming Medical University, Kunming, China

**Keywords:** metastatic breast cancer (mbc), childbearing age, females, nomogram, SEER, prognosis

## Abstract

**Introduction:**

Cancer in patients of childbearing age continues to become increasingly common. The purpose of this study was to explore the impact of metastatic breast cancer (MBC) on overall survival (OS) and cancer-specifific survival (CSS) in patients of childbearing age and to construct prognostic nomograms to predict OS and CSS.

**Methods:**

Data from MBC patients of childbearing age were obtained from the Surveillance, Epidemiology, and End Results (SEER) database between 2010 and 2015, and the patients were randomly assigned into the training and validation cohorts. Univariate and multivariate Cox analyses were used to search for independent prognostic factors impacting OS and CSS, and these data were used to construct nomograms. The concordance index (C-index), area under the curve (AUC), and calibration curves were used to determine the predictive accuracy and discriminative ability of the nomograms. Additional data were obtained from patients at the Yunnan Cancer Hospital to further verify the accuracy of the nomograms.

**Results:**

A total of 1,700 MBC patients of childbearing age were identifified from the SEER database, and an additional 92 eligible patients were enrolled at the Yunnan Cancer Hospital. Multivariate Cox analyses identifified 10 prognostic factors for OS and CSS that were used to construct the nomograms. The calibration curve for the probabilities of OS and CSS showed good agreement between nomogram prediction and clinical observations. The C-index of the nomogram for OS was 0.735 (95% CI = 0.725–0.744); the AUC at 3 years was 0.806 and 0.794 at 5 years.The nomogram predicted that the C-index of the CSS was 0.740 (95% CI = 0.730– 0.750); the AUC at 3 years was 0.811 and 0.789 at 5 years. The same results were observed in the validation cohort. Kaplan– Meier curves comparing the low-,medium-, and high-risk groups showed strong prediction results for the prognostic nomogram.

**Conclusion:**

We identifified several independent prognostic factors and constructed nomograms to predict the OS and CSS for MBC patients of childbearing age.These prognostic models should be considered in clinical practice to individualize treatments for this group of patients.

## Introduction

Breast cancer (BC) is the most frequent malignancy in women worldwide and remains the second leading cause of cancer-associated death in women (1, 2). Although the 5-year survival of BC is relatively high compared to other malignant tumors, distant metastasis remains a major cause of mortality. Previously, it has been reported that an increasing number of patients with metastatic breast cancer (MBC) are diagnosed at younger ages. Women of childbearing age tend to have an increased risk of disease progression (3). As women gradually postpone childbirth, the incidence of pregnancy-associated cancers has increased, causing a clinical challenge (4–6). There are distinct differences in the clinicopathologic characteristics and therapeutic strategies used in the management of cancers in women of childbearing age (7, 8). At childbearing age, the endocrine and reproductive functions of the ovaries reach a peak, modulating the secretion of hormones throughout the female body that may create a more oncogenic environment. Previous research has shown that the incidence of thyroid carcinoma in parturient women is higher compared to nulliparous women. These data are particularly important, as thyroid carcinoma has become the second most prevalent malignancy in women during pregnancy and during the reproductive stage (9). In addition, stage IV BC is a heterogeneous disease that is characterized by different metastatic sites, molecular subtypes, and diverse histopathologic features. Therefore, it is essential for MBC patients of childbearing age to have accurate predictions of outcomes and to define optimal treatment strategies.

Different treatment modalities are used in the management of patients at different physiological stages of life. For example, chemotherapy is effective in the treatment of stage IV BC, yet the adverse effects including amenorrhea and ovarian failure are more common in high-risk women of reproductive age (10). The development of personalized treatments requires more accurate risk estimation based on the specific clinicopathologic characteristics of patients.

Nomograms are accurate tools to predict cancer prognosis by quantifying individual risk based on clinicopathologic variables (11–13). Predicted individual survival results obtained from prognostic models can inform treatment selection. In a previous study, Zhao et al. (14) developed a nomogram to predict survival outcomes in MBC patients. However, to the best of our knowledge, nomograms to accurately predict the survival of MBC patients of childbearing age have not yet been established. This study screened the factors most associated with survival in women of childbearing age by performing univariate and multivariate Cox regression models. These data were integrated into a prognostic nomogram to predict the overall survival (OS) and cancer-specific survival (CSS) probabilities for MBC patients of reproductive age.

## Materials and methods

### Data sources and study design

The Surveillance, Epidemiology, and End Results (SEER) database stores a range of data concerning the current population demographics and provides access to specific information for related studies. The SEER database covers the majority of tumor types and collects cancer-related data including tumor characteristics, treatments, follow-up information, and the vital status of patients from different geographic areas for approximately 3 million patients (15).

The SEER*Stat software was used to extract data from MBC patients of childbearing age from 2010 to 2015. To identify suitable patients, the following inclusion criteria were set: 1) age ranging from 18 to 49 years at diagnosis, 2) female gender, 3) histology of infiltrating duct carcinoma, 4) BC diagnosed as the first and only primary tumor, 5) stage IV BC based on the American Joint Committee on Cancer (AJCC) TNM staging system, 6) positive pathological diagnosis, and 7) available follow-up information.

The exclusion criteria were as follows: 1) tumors of unknown differentiation grade, 2) unknown breast subtype, 3) undefined TNM staging, 4) unknown tumor sizes, 5) unknown races, 6) unknown marital status, and 7) unknown survival time. Data were also obtained from MBC patients of childbearing age who were initially diagnosed at the Yunnan Cancer Hospital between January 2012 and August 2016.

### Study variables

Patients were randomly assigned into the training and validation cohorts at a 7:3 ratio, and the relevant variables were extracted from the training cohort. Demographics included the age at diagnosis (18–30 years, 30–40 years, and 40–49 years), year of diagnosis (2010–2012 and 2013–2015), marital status (married and unmarried), and race (white, black, and others). Tumor characteristics included differentiation grade (grades I–IV), tumor size (<20 mm, 20–50 mm,and >50 mm), tumor location (central, upper, lower, axillary tail, and overlapping), laterality (left and right), molecular subtype (HR+/HER2-, HR+/HER2+, HR-/HER2+ and HR-/HER2-), TNM stage, and metastatic site (bone, brain, lung, and liver). Treatment modalities included surgery of the primary tumor (yes and no), chemotherapy (yes and no/unknown), and radiotherapy (yes and no/unknown).

### Development and validation of a predictive nomogram

Based on the results of the Cox regression analysis, two nomograms were developed to predict OS and CSS at 3 and 5 years. The concordance index (C-index) was used to assess the performances of these nomograms that refers to the proportion of all patient pairs whose predicted results were consistent with the actual results. Studies have defined the C-index thresholds for the predictive accuracy of nomograms as low (0.50–0.70), medium (0.71–0.90), and high accuracy (>0.90) (16). A bootstrapping method with 1,000 repetitions was performed to create calibration curves to validate the model in the training and validation cohorts. An extra external validation cohort from an independent group of Chinese patients was used to validate the accuracy and precision of the nomogram. Statistical analyses were carried out using R software (version 4.0.5).

Based on the analysis scores for each variable in the nomograms, the total points for all eligible patients from the SEER database were calculated. The patients were classified into low-, medium-, and high-risk groups based on the total scores of the nomogram using the X-Tile software. Kaplan–Meier analyses (including OS and CSS) and the log-rank test were used to test the accuracy of the prediction outcomes from the prognostic models.

### Follow-up

Data were retrospectively collected from MBC patients aged 18–49 years who were diagnosed between January 2012 and August 2016. The patients were followed up by telephone interviews, and the follow-up cutoff time was 1 June 2021 for the recording of survival outcomes (survival or death). The primary endpoints were OS and CSS. OS was defined as the time interval between the diagnosis of MBC and death or the date of the last follow-up. CSS was calculated as the time from the diagnosis to death from cancer.

### Statistical analyses

The categorical variables including demographics, tumor characteristics, and treatment were compared using a chi-square test. The primary endpoints were OS and CSS. The independent risk factors for OS and CSS were determined by univariate and multivariate Cox proportional risk regression models. The parameters refer to the hazard ratio (Hr) with a corresponding 95% confidence interval (CI). The analyses were carried out using SPSS software (version 26.0). The total score for each patient was calculated using R software (4.0.5, http://www.Rproject.org) based on the established Cox regression. The optimum cutoff value was selected using X-Tile (Version 3.6.1). The outcomes for low-, medium-, and high-risk patients were plotted using the Kaplan–Meier method and compared using a log-rank test. P-values of <0.05 for all of the variables were considered statistically significant.

## Results

### Patient characteristics

**Figure 1:**
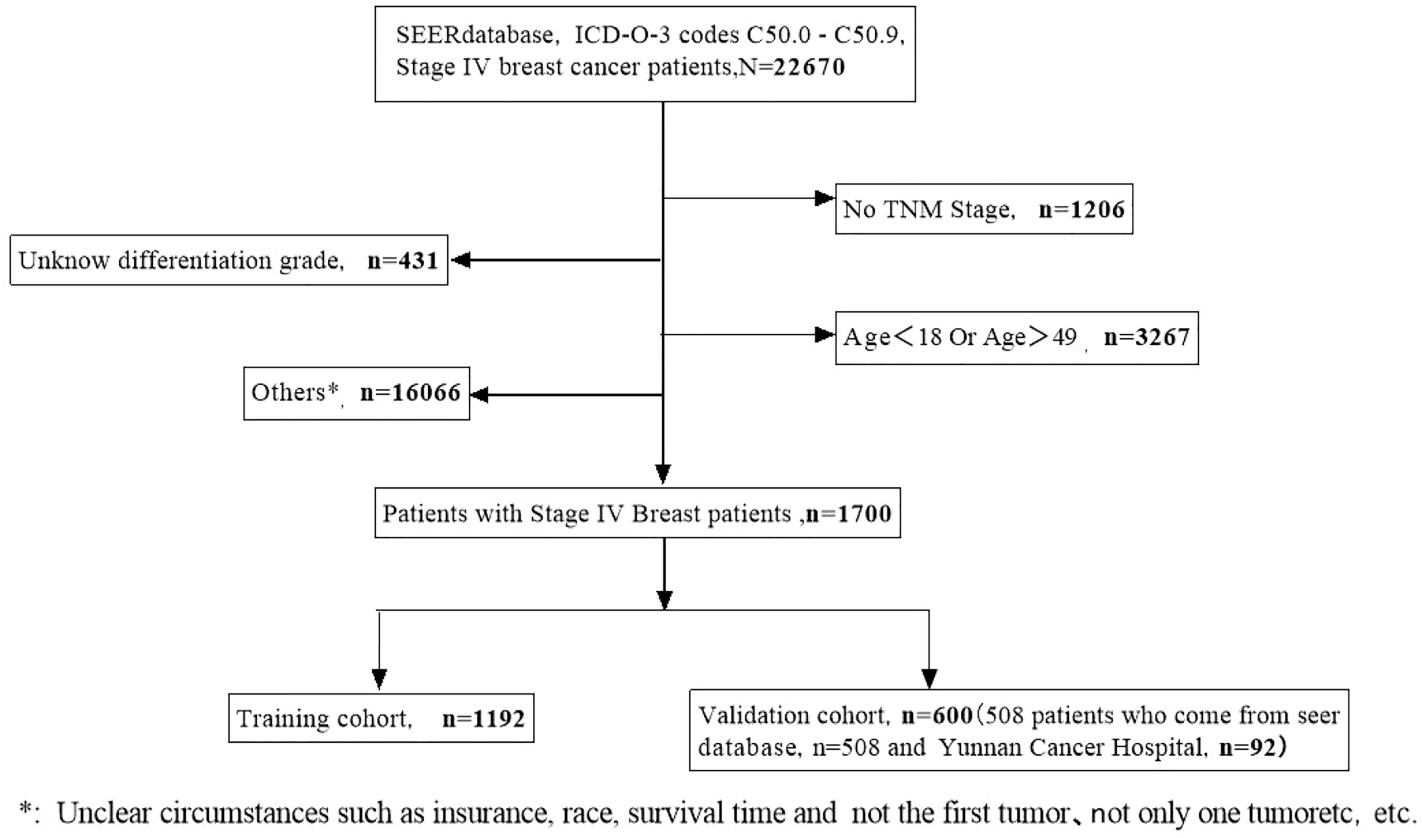
A flow diagram showing the screening process for the analysis of patients in the SEER and Yunnan cohorts.

Data from a total of 22,670 patients in the SEER database and an additional 92 eligible patients from Yunnan Cancer Hospital were analyzed in this study. After screening, 1,700 eligible patients met the inclusion criteria (**Figure 1**). Patients from the SEER database were randomly assigned to the training and validation cohorts at a ratio of 7:3. As presented in **Table 1**, there were 1,192 patients in the training cohort and 508 patients in the validation cohort. Most patients in the training cohort were 40–49 (59.2%) years old, 35.1% were 30–40 years old, and 5.7% were <30 years old. The majority of patients in both cohorts presented with bone metastasis. The most common BC subtype was luminal A (HR+/HER2-) disease, and the majority of patients received chemotherapy. Nearly half of the patients were married.

**Table 1:** Summary of the demographics and clinicopathologic characteristics of the patient cohorts.

Demographic or characteristic	Totaln=1,700	Training cohortn=1,192 (N%)	Validation cohortn=508 (N%)	P-value
**Age, years (N%)**				0.998
18-30	97	68 (5.7%)	29 (5.7%)	
30-40	597	418 (35.1%)	179 (35.2%)	
40-49	1,006	706 (59.2%)	300 (59.1%)	
**Year of diagnosis, N (%)**				0.133
2010-2012	826	565 (47.4%)	261 (51.4%)	
2013-2015	874	627 (52.6%)	247 (48.6%)	
**Race, N (%)**				0.072
White	1,147	823 (69%)	324 (63.8%)	
Black	365	248 (20.8%)	117 (23%)	
Other^1^	188	121 (10.2%)	67 (13.2%)	
**Laterality, N (%)**				0.808
Left	851	599 (50.3%)	252 (49.6%)	
Right	849	593 (49.7%)	256 (50.4%)	
**Location, N (%)**				0.132
Central^2^	99	70 (5.9%)	29 (5.7%)	
Upper	576	407 (34.1%)	169 (33.3%)	
Lower	184	134 (11.2%)	50 (9.8%)	
Axillary tail	7	4 (0.3%)	3 (0.6%)	
Overlapping	413	303 (25.4%)	110 (21.7%)	
Unknown	421	274 (23%)	147 (28.9%)	
**Grade, N (%)**				0.536
Well-differentiated; Grade I	74	47 (3.9%)	27 (5.3%)	
Moderately differentiated; Grade II	596	425 (35.7%)	171 (33.7%)	
Poorly differentiated; Grade III	1,022	715 (60%)	307 (60.4%)	
Undifferentiated; Grade IV	8	5 (0.4%)	3 (0.6%)	
**Tumor size (mm), N (%)**				0.278
<20	192	135 (11.3%)	57 (11.2%)	
20-50	659	476 (39.9%)	183 (36%)	
>50	849	581 (48.7%)	268 (52.8%)	
**T Stage, N (%)**				0.156
T_1_	188	133 (11.2%)	55 (10.8%)	
T_2_	692	493 (41.4%)	199 (39.2%)	
T_3_	373	271 (22.7%)	102 (20.1%)	
T_4_	447	295 (24.7%)	152 (29.9%)	
**N Stage, N (%)**				0.845
N_0_	274	198 (16.6%)	76 (15%)	
N_1_	872	610 (51.2%)	262 (51.6%)	
N_2_	251	175 (14.7%)	76 (15%)	
N_3_	303	209 (17.5%)	94 (18.5%)	
**Breast Surgery, N (%)**				0.111
No	870	595 (49.9%)	275 (54.1%)	
Yes	830	597 (50.4%)	233 (45.9%)	
**Chemotherapy, N (%)**				0.285
No	314	228 (19.1%)	86 (16.9%)	
Yes	1,386	964 (80.9%)	422 (83.1%)	
**Radiotherapy, N (%)**				0.131
No/Unknown	923	633 (53.1%)	290 (57.1%)	
Yes	777	559 (46.9%)	218 (42.9%)	
**Metastasis pattern, N%, Yes *vs*. No**				
Liver only	538	365 (30.6%)	173 (34.1%)	0.163
Brain only	98	70 (5.9%)	28 (5.5%)	0.770
Bone only	1,051	739 (62%)	312 (61.4%)	0.822
Lung only	423	292 (24.5%)	131 (25.8%)	0.573
**Breast subtype, N (%)**				0.862
HR+/HER2- (Luminal A)	829	582 (48.8%)	247 (48.6%)	
HR+/HER2+ (Luminal B)	389	268 (22.5%)	121 (23.8%)	
HR-/HER2+ (HER2-enriched)	204	142 (11.9%)	62 (12.2%)	
HR-/HER2- (Triple-Negative)	278	200 (16.8%)	78 (15.4%)	
**Marital status, N (%)**				0.437
Unmarried	762	527 (44.2%)	235 (46.3%)	
Married	938	665 (55.8%)	273 (53.7%)	

^1^ American Indian/AK Native, Asian/Pacific Islander.

^2^ Central portion of the breast or nipple. ^3^ Luminal A:ER(+),PR(+) and HER2(-) Luminal B:ER(+),PR(+) and HER2(+) HER2-enriched:ER(-),PR(-) and HER2(+) Triple-Negative:ER(-),PR(-) and HER2(-).

### Independent prognostic factors in metastatic breast cancer

**Figure 2:**
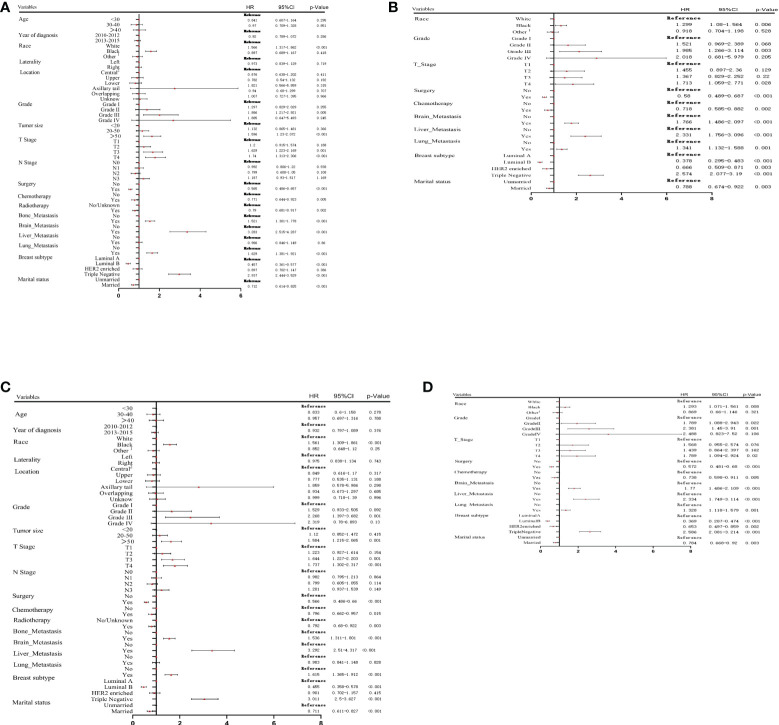
Univariate **(A)** and multivariate **(B)** Cox proportional hazards regression analysis of OS in the training cohort. Univariate **(C)** and multivariate **(D)** Cox proportional hazards regression analysis of CSS in the training cohort. 1: American Indian/AK Native, Asian/Pacific Islander; 2: central portion of breast or nipple.

In the training cohort, the results of univariate Cox analysis for OS showed that race, differentiation grade, tumor size, T stage, surgery, chemotherapy, radiotherapy, metastasis (liver, brain, and lung), BC subtype, and marital status were significant prognostic factors. These factors were then analyzed in multivariate Cox analysis. Our results showed that race, differentiation grade, T stage, surgery, chemotherapy, metastasis (liver, brain, and lung), BC subtype, and marital status were independent risk factors for OS in MBC patients (**Figures 2A, B**).

Univariate and multivariate Cox analyses were performed to screen the prognostic factors related to CSS. As presented in **Figures 2C, D**, race, differentiation grade, tumor size, T stage, surgery, chemotherapy, radiotherapy, metastasis (liver, brain, and lung), BC subtype, and marital status were significantly associated with CSS. Multivariate analysis showed that the independent prognostic factors for CSS included race, differentiation grade, T stage, surgery, chemotherapy, metastasis (liver, brain, and lung), BC subtype, and marital status.

### Prognostic nomogram for survival

**Figure 3:**
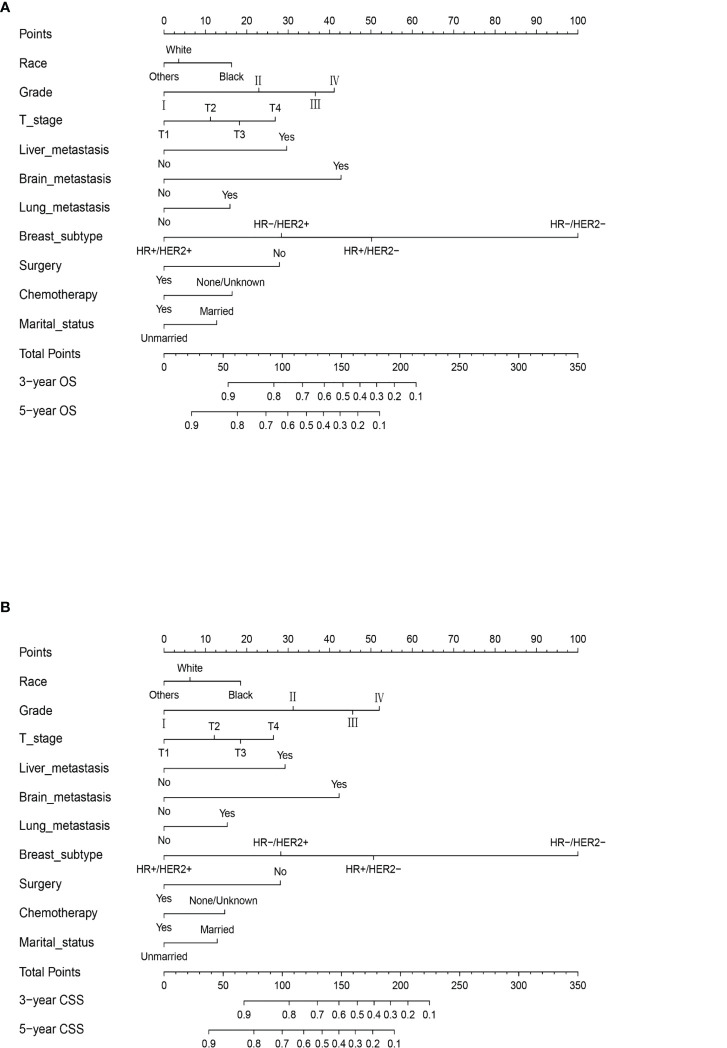
The prognostic nomograms for OS **(A)** and CSS **(B)** in MBC patients of childbearing age in the training cohort. Example of the nomogram. The nomogram can be used to calculate the prediction probability of OS and CSS. The nomogram shows the influence of different prediction variables. The influence of each variable is represented by the horizontal lines, with longer lines indicating a greater impact. The influence of each variable is visualized through multiple points on the corresponding horizontal line. By adding points related to each variable, the expected score size can be read on the response horizontal line at the bottom of the nomogram.

Data from the multivariate Cox regression analyses in the training cohort were used to develop predictive nomograms for OS and CSS at 3 and 5 years by integrating all of the independent prognostic factors. Both models indicated that the BC subtype had the largest impact on prognosis, followed by tumor grade and brain and liver metastases. Other factors included T stage, lung metastasis, surgery, chemotherapy, race, and marital status that had a moderate influence on OS and CSS. The specific scoring system for both nomograms is shown in **Figure 3**. Our nomogram can be interpreted, as each variable in the graph corresponded to a score according to the weight calculated by multivariate Cox regression analysis. The sum of the scores for all variables was used to give a total risk score for each patient to infer the OS and CSS. The specific methods for the nomogram interpretation have been previously reported (17).

In the calculation of the nomogram, for example, a black patient with poorly differentiated triple-negative BC classified as T2 who has received surgery and chemotherapy. The scores of each risk factor are black (10), married (0), poorly differentiated (40), triple-negative BC (100), T2 (10), surgery (0), and chemotherapy (0); so, the total score is 160. Our model predicts that the probability of the OS of patients at 3 years is 40% and the probability of OS at 5 years is 20%.

### Validation of the nomogram

**Figure 4:**
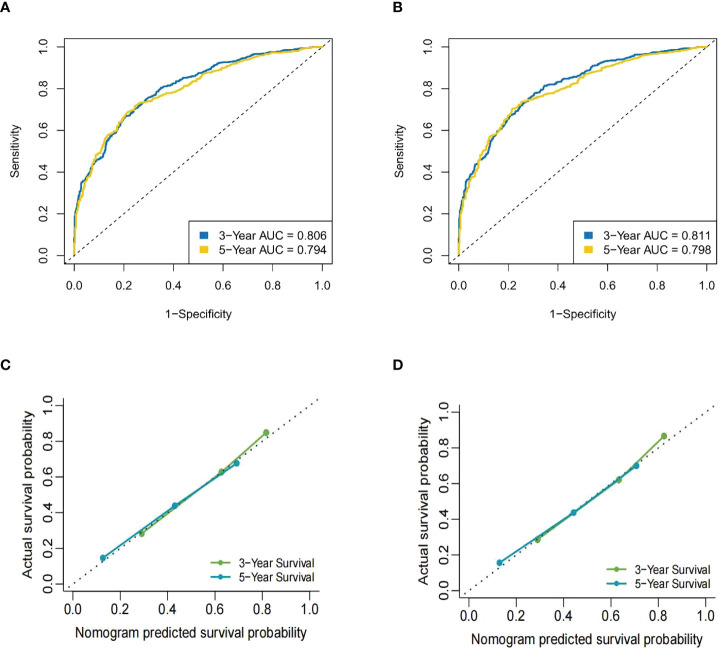
The ROC curve of the nomogram in the training cohort. **(A)** The AUC for OS at 3 years was 0.806 and at 5 years was 0.794. **(B)** The AUC for CSS at 3 years was 0.811 and at 5 years was 0.798. **(C)** The calibration curves for OS of the nomograms. **(D)** The calibration curves for CSS of the nomograms.

The C-index for OS predicted by the nomogram was 0.735 (95% CI = 0.725–0.744). The AUC [receiver operating characteristic (ROC) curve] at 3 years was 0.806 (95% CI: 0.78–0.83) and 0.794 at 5 years (95% CI: 0.76–0.82). The nomogram predicted that the C-index for CSS was 0.740 (95% CI = 0.730–0.750). The AUC (ROC curve) at 3 years was 0.811 (95% CI: 0.79–0.84) and at 5 years was 0.798 (95% CI: 0.77–0.83). These data showed that the nomograms were consistent between the predicted and actual survival of MBC patients of reproductive age (**Figure 4**). In the validation cohort, the predicted OS C-index was 0.710 (95% CI = 0.695–0.725). The AUC (ROC curve) at 3 years was 0.819 (95% CI: 0.8–0.83) and at 5 years was 0.789 (95% CI: 0.77–0.8). The nomogram predicted that the C-index of CSS was 0.712 (95% CI = 0.697–0.728). The AUC (ROC curve) at 3 years was 0.817 (95% CI: 0.79–0.83) and at 5 years was 0.786 (95% CI: 0.76–0.8). The data are presented in **Supplementary Figure S1**.

Data from the external validation cohort from Yunnan Cancer Hospital were used to further demonstrate the accuracy of the nomogram. In this group of patients, the C-index for OS was 0.721 (95% CI = 0.673–0.769) and for CSS was 0.712 (95% CI = 0.659–0.765).

### Kaplan–Meier Analysis

**Figure 5:**
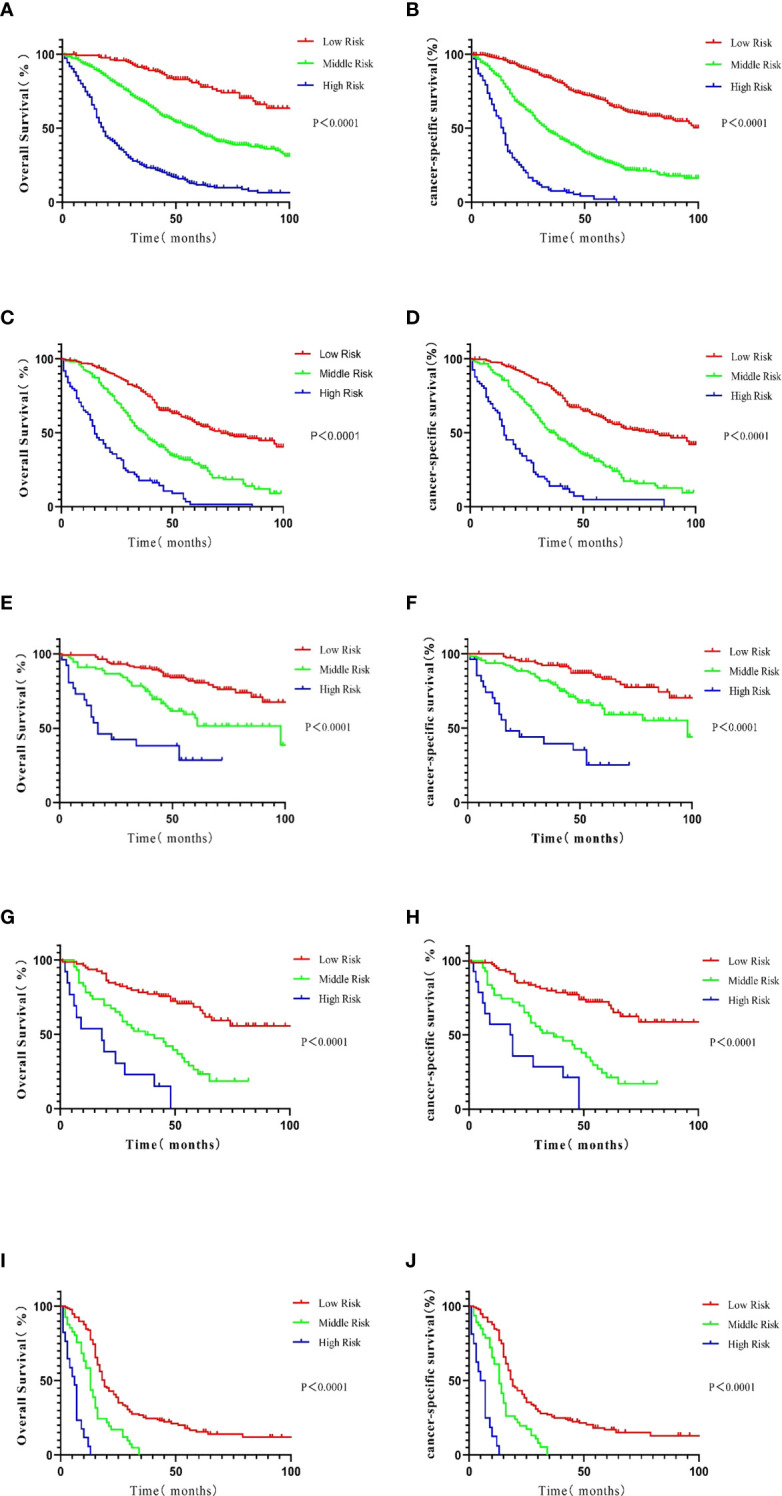
Kaplan–Meier analysis of patients in the training cohort. The survival curves were generated from the score calculated by the nomograph for OS **(A)** and CSS **(B)**. In patients with luminal A BC, the survival curve was generated from the score calculated by the nomograph: OS **(C)** and CSS **(D)**. In patients with the luminal B subtype, the survival curve was generated from the score calculated by the nomograph: OS **(E)** and CSS **(F)**. In patients with the HER2-enriched subtype, the survival curve was generated from the score calculated by the nomograph: OS **(G)** and CSS **(H)**. In patients with the triple-negative subtype, the survival curve was generated from the score calculated by the nomograph: OS **(I)** and CSS **(J)**.

To exclude the influence of different BC molecular subtypes (HR+/HER2-, luminal A; HR+/HER2+, luminal B; HR-/HER2+, HER2-enriched; HR-/HER2-, triple-negative) on the stability and accuracy of the model, Kaplan–Meier curves for OS were generated for low-, middle-, and high-risk patient groups in the training set, the validation set, and the four molecular subtypes. Specifically, the method further divided the patients from the training and validation sets and the samples from different subtypes into three subgroups (low-, medium-, and high-risk groups) according to the total score of the nomogram. These classifications were used to construct the survival curve. The same method was used in the analysis of CSS, and a Kaplan–Meier survival curve was generated based on data in the training dataset and the four molecular types after subgroup analysis (**Figure 5**). Data from the verification analysis are presented in the **Supplementary Material** and **Figure 2**.

After grouping according to the molecular classification of BC, the prognoses of patients in the low-, medium-, and high-risk subgroups in the survival curve based on the nomogram were significantly different (P < 0.0001), indicating good prediction capabilities of the nomogram.

X-Tile evaluates all possible divisions of data by dividing the data into several groups (18). Correlation can be calculated in each partition through various standard statistical tests. The program can select the highest χ^2^ values to determine the optimal segmentation of data. The calculations made by X-Tile were verified by StatView 5.0.1 (SAS Institute, Cary, NC, USA). We divided the OS and CSS of the training cohort according to the method described above. The risk score for OS in the low-risk group ranged from 426 to 498. The risk score in the medium-risk group ranged from 499 to 565, and the risk score in the high-risk group was >566. Similarly, for CSS, the risk score ranged from 406 to 527 in the low-risk group, 528 to 593 in the medium-risk group, and 594 to 693 in the high-risk group. Interestingly, most high-risk patients had triple-negative BC. The luminal B molecular subtype is essential in the low-risk population, and the other molecular types were attributed to the medium-risk population. The risk groups can be robustly divided according to molecular subtypes.

## Discussion

BC is the second-highest cause of cancer-related mortality in women and continues to pose a major threat to women’s health (19, 20). Women are capable of bearing children during a particular age range during which the endocrine and female reproductive systems are mature or fully functional. Breast development is a female secondary sexual characteristic that occurs in response to hormonal stimulation and results in the physiological characteristics of women of childbearing age (7, 8, 21). Studies have demonstrated that human cancers are controlled by hormones that are related to the metabolism of endogenous estrogens (22). In addition, it has been shown that young women and elderly patients have distinct changes in gene expression (23).

Nomograms are widely used in prediction models. Previously, Wang et al. (24) developed a prognostic nomogram for patients after bile duct surgery, and its performance was compared to conventional staging. The authors demonstrated that the nomogram was more accurate than conventional staging in predicting patient survival. Xie et al. (25) identified 56 differentially expressed mRNAs and determined that 26 of these differentially expressed genes were related to metastasis-free survival (MFS). Using these data, the authors developed a nomogram based on mRNA characteristics and clinical-related risk factors to predict the individual disease risk.

Several studies have taken similar approaches to developing risk prediction models (26, 27). We established a prognostic nomogram based on an analysis of the SEER database for OS and CSS in stage IV BC patients of childbearing age. Based on Cox regression, the nomogram could accurately predict OS and CSS at 3 and 5 years in stage IV BC patients. The primary cohort C-index (OS: 0.735, CSS: 0.74) and calibration curve indicated that the nomogram had a satisfactory performance. While previous prognostic nomograms have been developed in BC patients of childbearing age (28), the majority of research has been conducted in triple-negative BC. The data presented in this study are more extensive and cover all stage IV BC patients of childbearing age. We also selected patients of childbearing age from the Breast Department of Yunnan Cancer Hospital to obtain survival data at 3 and 5 years. These data were used to validate the OS and CSS nomograms with encouraging results (C-index OS: 0.721, CSS: 0.712).

In the nomogram, the variables affecting OS and CSS were the same. In the univariate Cox analysis of OS and CSS, race, tumor grade, tumor diameter, chemoradiotherapy, surgery, distant metastasis, BC cancer subtype, and marital status affected survival. In the multivariate Cox analysis, tumor diameter and radiotherapy did not affect OS and CSS. Gebski et al. (29) suggested that there was no direct evidence that radiotherapy is associated with OS in BC patients. Studies have suggested that tumor diameters <3 cm are generally considered a favorable prognostic factor (30). However, Mao et al. (31) suggested that tumor diameter is not an independent factor for OS and CSS, which was consistent with our findings.

Liu et al. (32) hypothesized that surgical resection of the primary tumor is beneficial to the survival of new stage IV BC patients and developed a nomogram to identify patients who could benefit from primary tumor resection. The study included 13 factors [e.g., race, cohabitation, tumor grade, tissue type, molecular subtype, metastasis (brain, liver, and lung), and chemotherapy] that were mostly consistent with our results. These data further support the validity of our findings. In addition, Mou et al. (33) showed that some clinical features and serological markers (pathological type of disease, multiple bone metastases, organ metastasis, and serum lactate dehydrogenase levels) can predict the OS in patients with metastatic BC. Pathological type, multiple bone metastases, and organ metastasis were consistent with our findings.

Related studies have demonstrated that chemotherapy plays a significant role in treating the large tumor burden, lymph node invasion, or recurrent/metastatic BC. Marital status was also an independent risk factor for patient prognosis. Tao et al. (34) analyzed patients with metastatic bladder cancer based on marital status and showed that while marital status had little impact on OS, it was an independent prognostic factor (34, 35). These data may be explained as married patients may prefer to accept and cooperate during treatment. Ethnicity was also included in the nomogram. Studies have shown that black women have a poor prognosis due to their biological differences and socioeconomic status, and even geographical factors and national policies (36–38).

BC is divided into four subtypes, specifically, luminal A, luminal B, HER2-enriched, and triple-negative forms. From an epidemiological perspective, patients with luminal A BC are usually older than those who have the other subtypes and the prognosis for luminal A disease is better. However, according to Belhadj et al. (39), in western Algeria, younger (<40 years) and intermediate-age patients (41–54 years) were most likely to have luminal A BC, whereas older patients had triple-negative disease and had the highest mean disease-free survival (DFS). Young women are generally considered as having HER2+ or triple-negative molecular subtypes and may have a poor prognosis. A study in China by Li et al. (40) found that younger patients (<35 years) tended to have larger tumors, positive lymph nodes, higher histological grades, non-luminal type disease, higher Ki67 expression, and poorer prognosis. Patients with triple-negative tumors were the youngest (mean 48.4 years) and had the greatest proportion of grade 3 histology and a poor prognosis. Therefore, there is a need to study this particular group of patients at reproductive age. Unfortunately, however, no reproductive information was retrieved from the SEER database or the external validation cohort, and further data are needed to verify the robustness of our model.

While our study provides intriguing results, it has several limitations. Firstly, our study was conducted using data from the SEER database in which most patients (69%) were Caucasian and may be genetically different from Chinese patients. Secondly, the SEER data and validation in Yunnan Cancer Hospital were retrospectively analyzed and subject to bias. Thirdly, the SEER database did not provide clear information on other patients and treatment methods such as smoking, related serum markers, chemotherapy regimens, targeted therapies, and endocrine therapies. Finally, the SEER database did not capture data relating to the fertility of the patients. In future studies, we will focus on collecting more clinical information to update our nomogram and guide clinical treatments. Our nomogram can still be used to guide personalized risk prediction and the staging of metastatic BC patients of childbearing age.

## Data availability statement

The datasets presented in this study can be found in online repositories. The names of the repository/repositories and accession number(s) can be found below: https://seer.cancer.gov/.

## Ethics statement

The studies involving human participants were reviewed and approved by Ethics Committee of Yunnan Cancer Hospital. The patients/participants provided their written informed consent to participate in this study.

## Author contributions

XM wrote the main manuscript text. JB designed the project. YX, ZL, SQ and WW prepared statistical analysis, tables and figures. All authors contributed to the article and approved the submitted version.

## Conflict of interest

The authors declare that the research was conducted in the absence of any commercial or financial relationships that could be construed as a potential conflict of interest.

## Publisher’s note

All claims expressed in this article are solely those of the authors and do not necessarily represent those of their affiliated organizations, or those of the publisher, the editors and the reviewers. Any product that may be evaluated in this article, or claim that may be made by its manufacturer, is not guaranteed or endorsed by the publisher.

## References

[1] Siegel RL Miller KD Jemal A. Cancer statistics, 2018. CA: A Cancer Journal for Clinicians (2018) 68(1):7–30. doi: 10.3322/caac.21442 29313949

[2] Ferlay J Soerjomataram I Dikshit R Eser S Mathers C Rebelo M. Cancer incidence and mortality worldwide: sources, methods and major patterns in GLOBOCAN 2012. Int J Cancer (2015) 136(5):E359–86. doi: 10.1002/ijc.29210 25220842

[3] DeSantis CE Fedewa SA Sauer AG Kramer JL Smith RA Ahmedin J. Breast cancer statistics, 2015: Convergence of incidence rates between black and white women. CA: Cancer J Clin (2016) 66:31–42. doi: 10.3322/caac.21320 26513636

[4] Amant F Halaska MJ Fumagalli M Lok C Calstere KV. Gynecologic cancers in pregnancy: guidelines of a second international consensus meeting. Int J Gynecol Cancer (2014) 24:394–403. doi: 10.1097/IGC.0000000000000062 24445819

[5] Dotters-Katz S McNeil M Limmer J Kuller J. Cancer and pregnancy: the clinician's perspective. Obstet Gynecol Surv (2014) 69:277–86. doi: 10.1097/OGX.0000000000000068 25101693

[6] Salani R Billingsley CC Crafton SM. Cancer and pregnancy: an overview for obstetricians and gynecologists. Am J Obstet Gynecol (2014) 211:7–14. doi: 10.1016/j.ajog.2013.12.002 24316272

[7] Liedtke C Hess KR Karn T Rody A Kiesel L Hortobagyi GN. The prognostic impact of age in patients with triple-negative breast cancer. Breast Cancer Res Treat (2013) 138(2):591–9. doi: 10.1007/s10549-013-2461-x 23460246

[8] Plichta JK Thomas SM Vernon R Fayanju OM Rosenberger LH Hyslop T. Breast cancer tumor histopathology, stage at presentation, and treatment in the extremes of age. Breast Cancer Res Treat (2020) 180(1):227–35. doi: 10.1007/s10549-020-05542-4 PMC7066434 31980967

[9] Smith LH Danielsen B Allen ME Cress R. Cancer associated with obstetric delivery: results of linkage with the California cancer registry. Am J Obstet Gynecol (2003) 189(4):1128–35. doi: 10.1067/S0002-9378(03)00537-4 14586366

[10] Simsek FS Balci TA Donder Y Ugur K Kilinc F. How important is the timing of radioiodine ablation in differentiated thyroidal carcinomas: A referral centre experience. Rev Esp Med Nucl Imagen Mol (Engl Ed) (2020) 39(3):157–62. doi: 10.1016/j.remnie.2019.08.003 31982352

[11] Wang J Wu Y He W Yang B Gou X. Nomogram for predicting overall survival of patients with bladder cancer: A population-based study. Int J Biol Markers (2020) 35(2):29–39. doi: 10.1177/1724600820907605 32312147

[12] Cao J Yuan P Wang L Wang YQ Ma HH Yuan XS. Clinical nomogram for predicting survival of esophageal cancer patients after esophagectomy. Sci Rep (2016) 6:26684. doi: 10.1038/srep26684 27215834 PMC4877645

[13] Li Y Ju J Liu X Gao T Wang ZD Ni QW. Nomograms for predicting long-term overall survival and cancer-specific survival in patients with major salivary gland cancer: a population-based study. Oncotarget (2017) 8(15):24469–82. doi: 10.18632/oncotarget.14905 PMC5421863 28160551

[14] Zhao W Wu L Zhao A Tian Q Shen YW Wang F. A nomogram for predicting survival in patients with *de novo* metastatic breast cancer: a population-based study. BMC Cancer (2020) 20(1):982. doi: 10.1186/s12885-020-07449-1 33046035 PMC7549197

[15] Cronin KA Ries LA Edwards BK. The surveillance, epidemiology, and end results (SEER) program of the national cancer institute. Cancer (2014) 120(suppl 23):3755–7. doi: 10.1002/cncr.29049 25412387

[16] Harrell F Jr Lee KL Mark DB. Multivariable prognostic models: issues in developing models, evaluating assumptions and adequacy, and measuring and reducing errors. Stat Med (1996) 15(4):361–87. doi: 10.1002/(SICI)1097-0258(19960229)15:4<361::AID-SIM168>3.0.CO;2-4 8668867

[17] Iasonos A Schrag D Raj GV Panageas KS. How to build and interpret a nomogram for cancer prognosis. J Clin Oncol (2008) 26(8):1364–70. doi: 10.1200/JCO.2007.12.9791 18323559

[18] Camp RL Dolled-Filhart M Rimm DL. X-Tile: a new bio-informatics tool for biomarker assessment and outcome-based cut-point optimization. Clin Cancer Res (2004) 10(21):7252–9. doi: 10.1158/1078-0432.CCR-04-0713 15534099

[19] Siegel RL Miller KD Jemal A. Cancer statistics, 2017. CA Cancer J Clin (2017) 67(1):7–30. doi: 10.3322/caac.21387 28055103

[20] Chen W Zheng R Baade PD Zhang SW Zeng HM Bray FD. Cancer statistics in China, 2015. CA Cancer J Clin (2016) ; 66(2):115–32. doi: 10.3322/caac.21338 26808342

[21] Aapro M Wildiers H. Triple-negative breast cancer in the older population. Ann Oncol (2012) Suppl 6:vi52–5. doi: 10.1093/annonc/mds189 23012304

[22] Yao J Fang L-C Yang Z-L Huang H Li Y Deng J. Mixed lineage leukaemia histone methylases 1 collaborate with ERα to regulate HOXA10 expression in AML. Biosci Rep (2014) 34(6):e0015. 10.1042/BSR20140116 PMC4266925 25307539

[23] Anders CK Hsu DS Broadwater G Acharya CR Foekens JA Zhang Y. Young age at diagnosis correlates with worse prognosis and defines a subset of breast cancers with shared patterns of gene expression. J Clin Oncol (2008) 26(20):3324–30. doi: 10.1200/JCO.2007.14.2471 18612148

[24] Wang Y Li J Xia Y Gong RY Wang K Yan ZL. Prognostic nomogram for intrahepatic cholangiocarcinoma after partial hepatectomy. J Clin Oncol (2013) 31(9):1188–95. doi: 10.1200/JCO.2012.41.5984 23358969

[25] Xie X Wang J Shi D Zou YT Xiong ZC Li X. Identification of a 4-mRNA metastasis-related prognostic signature for patients with breast cancer. J Cell Mol Med (2019) 23(2):1439–47. doi: 10.1111/jcmm.14049 PMC6349190 30484951

[26] Sternberg CN. Are currently available nomograms better than stage groupings for bladder cancer? J Clin Oncol (2006) 24:3819–20. doi: 10.1200/JCO.2006.07.1290 16864852

[27] Touijer K Scardino PT. Nomograms for staging, prognosis, and predicting treatment outcomes. Cancer (2009) 115:3107–11. doi: 10.1002/cncr.24352 19544538

[28] Cui X Song D Li X. Construction and validation of nomograms predicting survival in triple-negative breast cancer patients of childbearing age. Front Oncol (2021) 10:636549. doi: 10.3389/fonc.2020.636549 33628740 PMC7898905

[29] Gebski Val Lagleva Marivic Anthony Keech Simes John. Survival effects of postmastectomy adjuvant radiation therapy using biologically equivalent doses: a clinical perspective. J Natl Cancer Instr (2006) 98(1):26–38. doi: 10.1093/jnci/djj002 16391369

[30] Lee JI Kim JK Kim Do Y Ahn SH Park JY Kim SU. Prognosis of hepatocellular carcinoma patients with extrahepatic metastasis and the controllability of intrahepatic lesions. Clin Exp Metas. (2014) 31:475–82. doi: 10.1007/s10585-014-9641-x 24496959

[31] Mao K Yan Y Zhang J Wang J Wang RM Ling XJ. The impact of liver resection on survival outcomes of hepatocellular carcinoma patients with extrahepatic metastases: A propensity score matching study. Cancer Med (2018) 7(9):4475–84. doi: 10.1002/cam4.1738 PMC6143947 30117307

[32] Liu X Wang C Feng Y Shen CY He T Wang Z. The prognostic role of surgery and a nomogram to predict the survival of stage IV breast cancer patients. Gland Surg (2022) 11(7):1224–39. doi: 10.21037/gs-22-330 PMC9346227 35935562

[33] Mou H Wang Z Zhang W Li GQ Zhou H Wang EY. Clinical features and serological markers risk model predicts overall survival in patients undergoing breast cancer and bone metastasis surgeries. Front Oncol (2021) 11:693689. doi: 10.3389/fonc.2021.693689 34604031 PMC8484887

[34] Tao L Pan X Zhang L Wang JW Zhang ZC Zhang L. Marital status and prognostic nomogram for bladder cancer with distant metastasis: A SEER-based study. Front Oncol (2020) 10:586458. doi: 10.3389/fonc.2020.586458 33194738 PMC7654226

[35] Sammon JD Morgan M Djahangirian O Trinh QD Sun M Ghani KR. Marital poorer status: a gender-independent risk factor for survival after radical cystectomy. BJU Int (2012) 110:1301–9. doi: 10.1111/j.1464-410X.2012.10993.x 22449122

[36] Jemal A Robbins AS Lin CC Flanders WD DeSantis CE Ward EM. That factors contributed to black-white disparities in survival nonelderly women with breast cancer between 2004 and 2013. J Clin Oncol (2018) 36(1):14–24. doi: 10.1200/JCO.2017.73.7932 29035645

[37] Jatoi I Anderson WF Rao SR Devesa SS. Breast cancer trends among black and white women in the united states. J Clin Oncol (2005) 23(31):7836–41. doi: 10.1200/JCO.2004.01.0421 16258086

[38] Newman LA Griffifith KA Jatoi I Simon MS Crowe JP Colditz GA. Metaethnicity analysis of survival in African American and white American patients with breast cancer: ethnicity compared with socioeconomic status. J Clin Oncol (2006) 24(9):1342–9. doi: 10.1200/JCO.2005.03.3472 16549828

[39] Belhadj A Seddiki S Belhadj A Zakmout B Kader Amine Araba AE Sahraoui T. Prevalence and prognosis of molecular phenotypes in breast cancer patients by age: a population-based retrospective cohort study in western Algeria. Pan Afr Med J (2021) 38:88. doi: 10.11604/pamj.2021.38.88.21370 33889254 PMC8033184

[40] Li S Wang X Yang J Lv M Zhang X Li CL. Clinicopathological features and survival of early stage breast cancer in northwest China: A population-based retrospective study of 1287 patients. Thorac Cancer (2018) 9(1):10–8. doi: 10.1111/1759-7714.12503 PMC5754296 28976077

