# Supplementary material for: Construction and validation of a prognostic nomogram in metastatic breast cancer patients of childbearing age: A study based on the SEER database and a Chinese cohort
Source: Front Oncol. 2022 Nov 25;12:999873. doi: 10.3389/fonc.2022.999873 (PMC9732809; doi:10.3389/fonc.2022.999873)
Supplement: Supplementary file 1 [file DataSheet_1.pdf]

A

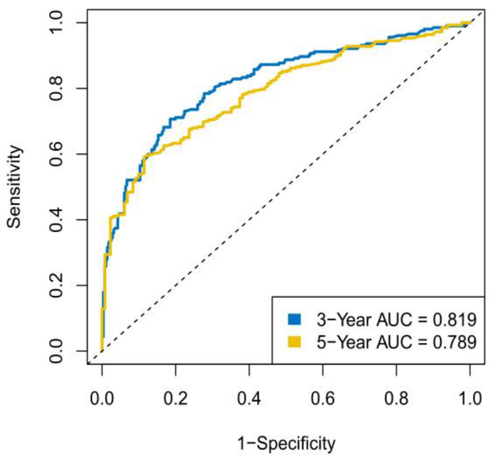

B

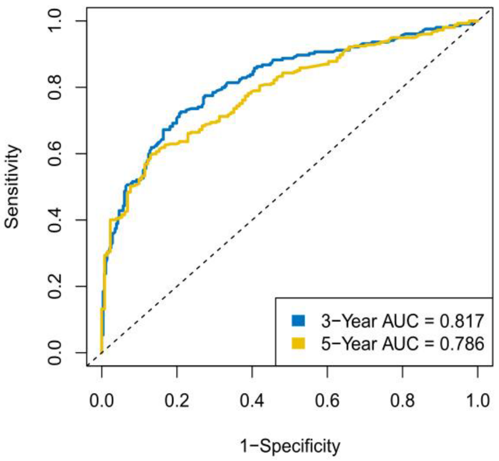

C

D

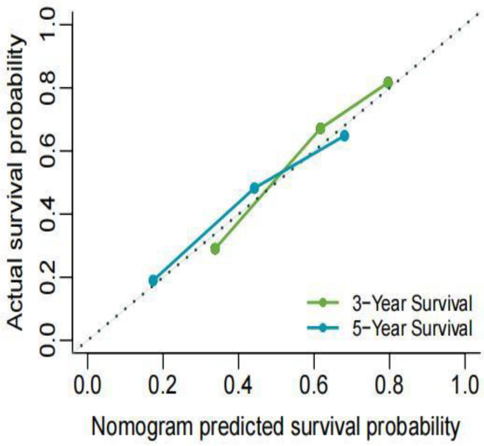

Supplement Figure. 1 ROC curve of the nomogram in the Validation cohort. A:The AUC in 3-years for OS was 0.819 and 5-years was 0.789 in the Training cohort. B:The AUC in 3-years for CSS was 0.817 and 5-years was 0.786 in the Training cohort. C:Calibration curves of the nomograms for OS. D: Calibration curves of the nomograms for CSS.

A

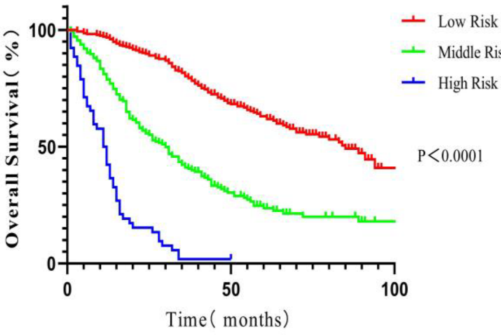
Overall Survival（ % ）
100
50
0
0
50
100
Time（ months）
Low Risk
Middle Risk
High Risk
P＜0.0001

B

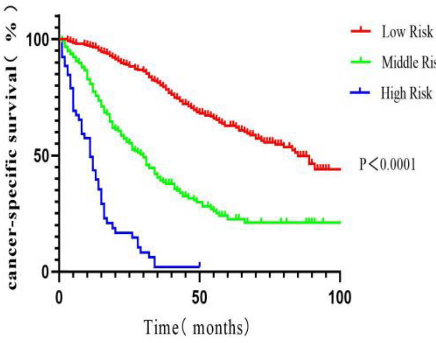
cancer-specific survival（ % ）
100
50
0
0
50
100
Time（ months）
Low Risk
Middle Risk
High Risk
P＜0.0001

C

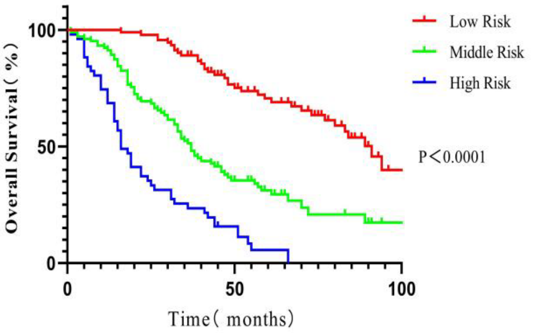
Overall Survival（ % ）
100
50
0
0
50
100
Time（ months）
Low Risk
Middle Risk
High Risk
P＜0.0001

D

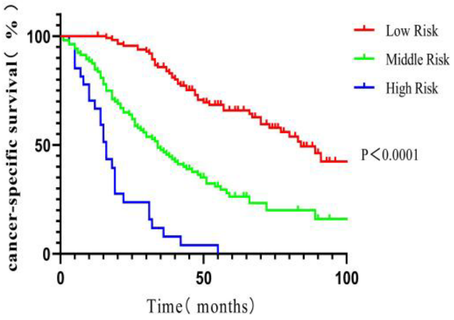
cancer-specific survival（ % ）
100
50
0
0
50
100
Time（ months）
Low Risk
Middle Risk
High Risk
P＜0.0001

E

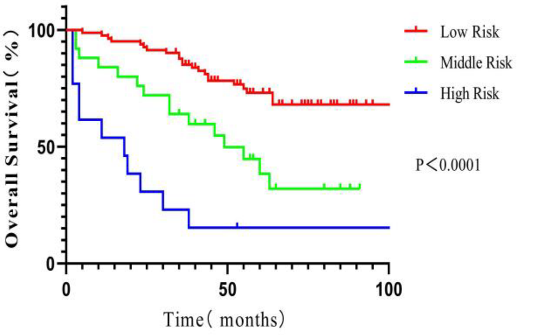
Overall Survival（ % ）
100
50
0
0
50
100
Time（ months）
Low Risk
Middle Risk
High Risk
P＜0.0001

F

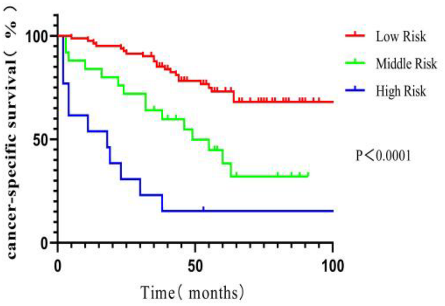
cancer-specific survival（ % ）
100
50
0
0
50
100
Time（ months）
Low Risk
Middle Risk
High Risk
P＜0.0001

G

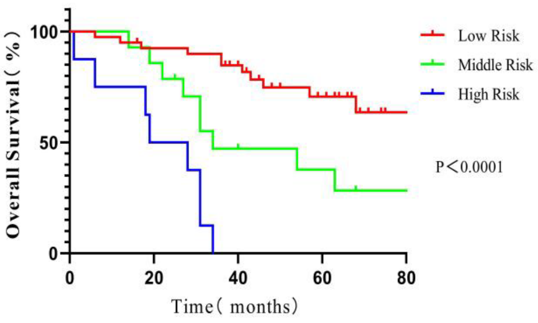
Overall Survival（ % ）
100
50
0
0
20
40
60
80
Time（ months）
Low Risk
Middle Risk
High Risk
P＜0.0001

H

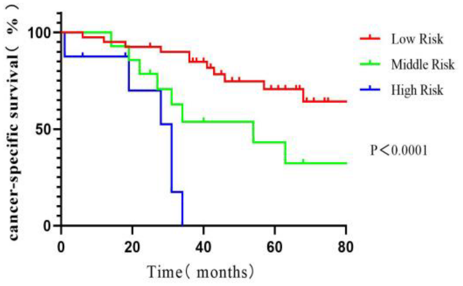
cancer-specific survival（ % ）
100
50
0
0
20
40
60
80
Time（ months）
Low Risk
Middle Risk
High Risk
P＜0.0001

I

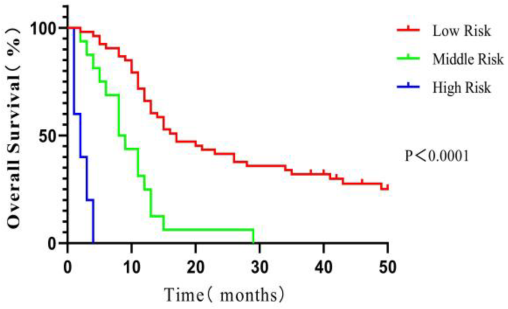

J

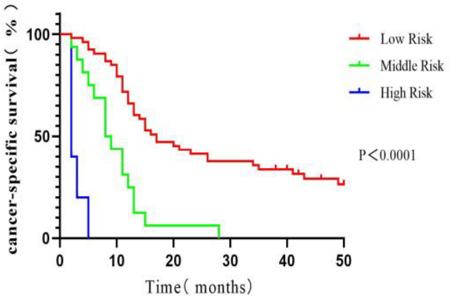

Supplement_Figure 2 Kaplan – Meier curves of Validation cohort . The survival curve is drawn by the score calculated by the nomograph in the **Validation cohort**: OS (A) and CSS (B) ; In **Luminal A** subtype, the survival curve was drawn by the score calculated by nomograph: OS (C) and CSS (D)；In **Luminal B** subtype, the survival curve was drawn by the score calculated by nomograph: OS (E) and CSS (F)；In **HER2 enriched** subtype, the survival curve was drawn by the score calculated by nomograph: OS (G) and CSS (H)；In **Triple Negative subtype**, the survival curve was drawn by the score calculated by nomograph: OS (I) and CSS (J).
